# Overexpression of alcohol dehydrogenase 1 A inhibits the progress of triple negative breast cancer via Wnt/β-catenin signaling

**DOI:** 10.1038/s41598-025-17643-5

**Published:** 2025-09-26

**Authors:** Lihong Su, Chunlin Qiao, Jin Luo, Bianling Zhu, Yunxiao Liu

**Affiliations:** 1 Department of Pathology, Shanxi Provincial People’s Hospital, Taiyuan, 030000 China; 2 Department of Pathology, Shanxi Provincial People’s Hospital, No. 29, Shuangtasi Street, Taiyuan, 030000 Shanxi China

**Keywords:** Breast cancer, Prognosis, Tyrosine metabolism, Migration, Proliferation, Breast cancer, Mesenchymal migration, Oncogenesis

## Abstract

The identification of novel therapeutic targets in triple negative breast cancer (TNBC) continues to be of paramount importance. In this context, ADH1A (Alcohol Dehydrogenase 1 A), a protein involved in tyrosine metabolism, was comprehensively examined to assess its expression and functional roles in TNBC. A combination of bioinformatics approaches and local tissue analyses was utilized to determine the expression levels of ADH1A in TNBC samples. Genetic manipulation techniques were employed to alter ADH1A expression, and the subsequent effects on TNBC cell behavior were systematically analyzed. This study is the first to report on the alterations of 14 genes related to tyrosine metabolism within the TCGA-TNBC cohorts. Notably, reduced expression of these enzymes is associated with poorer survival outcomes in patients with TNBC. An analysis of the TCGA database revealed reduced levels of ADH1A in human TNBC tissues. Furthermore, ADH1A protein expression was diminished in TNBC tissues of patients who received local treatment, in contrast to the elevated expression observed in adjacent normal tissues. In the MDA-MB-231 and SUM159PT cell lines, ADH1A knockdown significantly promoted cell proliferation, migration, and invasion. On the contrary, ADH1A overexpression inhibited cell proliferation, migration, and invasion, while inducing cell apoptosis. Mechanistically, the overexpression of ADH1A may attenuate the malignant characteristics of TNBC cells by inhibiting the Wnt/β-catenin signaling pathway. In conclusion, ADH1A may be a useful biomarker for TNBC prognosis. This study is the first to reveal that ADH1A inhibits the malignant progression of TNBC via the Wnt/β-catenin signaling pathway.

**Supplementary Information:**

The online version contains supplementary material available at 10.1038/s41598-025-17643-5.

## Introduction

Breast cancer (BRCA) is the most prevalent malignancy worldwide and constitutes the primary cause of cancer-related morbidity and mortality among women^1^. The pathogenesis and precise etiology of breast cancer remain incompletely understood, with numerous high-risk factors implicated in its development. Furthermore, breast cancer encompasses multiple subtypes, among which triple negative breast cancer (TNBC) represents the most lethal, accounting for 15–20% of all cases^2^. Despite advancements in therapeutic interventions, treatment options for TNBC remain constrained, frequently leading to an unfavorable prognosis^3^. Therefore, identifying new therapeutic targets for TNBC is crucial for improving clinical management.

Tyrosine, a vital aromatic amino acid for protein synthesis in all organisms, also acts as an alternative energy source, supporting various biochemical functions. Disruptions in tyrosine metabolism have been implicated in the pathogenesis of various cancers, such as gastroesophageal cancer^4^ and pulmonary carcinoma^5^. However, the molecular alterations and profiles associated with tyrosine catabolism in TNBC progression remain poorly understood. Recent evidence suggests a significant interaction between tyrosine metabolism and characterization of the tumor microenvironment infiltration^6^. Here, we performed a comprehensive analysis of TNBC datasets from The Cancer Genome Atlas (TCGA) to investigate the expression patterns of tyrosine metabolism-related genes (TRGs). Our findings revealed that ADH1A (Alcohol Dehydrogenase 1 A), a protein involved in tyrosine metabolism, is significantly downregulated in TNBC tissues and is associated with poor prognosis in TNBC patients.

Building on these results, we further examined the expression of ADH1A and its potential role and mechanisms of action in TNBC, aiming to elucidate the functional significance and molecular underpinnings of this protein in the progression of TNBC.

## Materials and methods

### Data acquisition

We obtained gene expression, prognostic, and clinicopathological data related to breast cancer from The Cancer Genome Atlas (TCGA) database, utilizing 1,198 BRCA samples for subsequent analyses. RNA-seq data from normal breast tissues and TNBC samples were obtained from three independent data sets from Gene Expression Omnibus (GEO) database (GSE38959, GSE45827 and GSE65194)^7,8^. In addition, another data set (GSE76124) with large numbers of TNBC samples was sued to analyze the correlation between ADH1A and epithelial-mesenchymal transition (EMT) markers. Each dataset underwent normalization and log2 transformation to identify differentially expressed genes (DEGs), which were subsequently visualized using volcano plots. The criteria for filtering DEGs were set as follows: |log2FC| ≥ 1 and an adjusted P-value (adj. P) < 0.05. Additionally, we extracted a total of 42 tyrosine metabolism related genes (TRGs) from the Molecular Signatures Database (MSigDB) Hallmark gene set ‘KEGG_TYROSINE_METABOLISM’ (hsa00350)^9^. All experiments were performed in accordance with the relevant guidelines.

### Clustering analysis

An intersection of identified DEGs with the Gene set revealed 14 overlapping TRGs. These TRGs were clustered using the “Consensus Cluster Plus” R package with Euclidean distance and K-means, allowing up to five clusters for stable classification. This process identified two subtypes, and differential gene expression between them was analyzed, using criteria of absolute log2FC > 1 and *P* < 0.05.

### Functional enrichment analysis

The DAVID Bioinformatics Resources (https://david.ncifcrf.gov/) was employed to identify and enhance the biological characteristics, including biological processes, cellular components, molecular functions, and pathways, of key differentially expressed genes (DEGs). The VennDiagram package was used to present significant co-expression genes. To investigate the mechanisms underlying the two tyrosine metabolism subtypes associated with TNBC, we conducted Gene Ontology (GO) enrichment analysis and Kyoto Encyclopedia of Genes and Genomes (KEGG) pathway analysis^10^ using the “clusterProfiler” R package. A significance threshold of *P* < 0.05 was established for enrichment analysis.

### Identification the key genes of TRGs in TNBC

Least Absolute Shrinkage and Selection Operator (LASSO) regression is a penalized regression technique employed for variable selection in high-dimensional datasets to construct prognostic models. In this study, we identified significant genes related to tyrosine metabolism from TNBC specimens and conducted optimal cutoff analysis utilizing the “surv_cutpoint” function from the “survminer” R package. Following this, we applied the LASSO method within a Cox regression framework to select the most informative prognostic genes, using the “glmnet” R package, based on the optimal cutoff risk score. TNBC Patients were divided into high-risk and low-risk groups and then subjected to the Kaplan-Meier (KM) survival analysis.

### Analysis of ADH1A expression

The expression of ADH1A was analyzed to compare its differential expression between triple-negative breast cancer (TNBC) tissues and normal tissues utilizing the UALCAN platform (https://ualcan.path.uab.edu/index.html). TNBC Patients were divided into high-level and low-level ADH1A mRNA groups and then subjected to the Kaplan-Meier (KM) survival analysis (https://kmplot.com/analysis).

### Patients and tissue samples

TNBC tissues and corresponding adjacent normal breast tissues were obtained from patients undergoing surgical procedures at the Breast Cancer Surgery Department of the Shanxi Provincial People’s Hospital. None of the patients had received any form of therapy prior to surgery. The tissue samples were collected immediately following tumor excision and preserved at −80 °C. All participants provided informed consent. This study was approved by the Ethics Committee of Shanxi Provincial People’s Hospital.

### Pathological staining

To investigate the expression of ADH1A through immunohistochemical (IHC) staining, we employed a tissue microarray comprising 140 breast cancer tissue samples and 77 adjacent normal tissue samples. The IHC staining procedure adhered to established protocols, utilizing anti-ADH1A antibodies (Abcam; ab239875). The stained sections were subsequently mounted and examined using a Nikon microscope.

### Cell lines and culture

The breast cancer cell lines MDA-MB-231 and SUM159PT were obtained from the American Type Culture Collection (ATCC). MDA-MB-231 cells were maintained in Leibovitz’s L15 medium supplemented with 10% fetal bovine serum (FBS), whereas SUM159PT cells were cultured in RPMI-1640 medium supplemented with 10% FBS and 1% penicillin/streptomycin. Both DMEM and RPMI-1640 media were procured from Hyclone. To study the effects of Wnt signaling on cell malignant behavior, cells were treated with 10 µM Laduviglusib (a potent Wnt/β-catenin signaling pathway activator; HY-10182; MedChemExpress, China) for 24 h^11^.

### Lentivirus packaging and infection

Lentiviral vectors, specifically pGLV3/CMV/GFP-Puro, were procured from GenePharma, located in Shanghai, China. The production of the pLV3-ADH1A and control lentiviruses was carried out following established protocols. Subsequently, cells were transduced with the pLV3-vector and pLV3-ADH1A lentiviruses. Following thorough mixing, the cells were incubated for another 48 h, after which they were collected for western blot analysis.

### Silencing of the ADH1A gene by SiRNA in TNBC cells

Specific siRNAs targeting ADH1A mRNA were designed and procured from GenePharma (Suzhou, China). A scrambled siRNA from GenePharma, which does not target any gene, was used as a negative control. The sequences of the siRNA were as follows: sense strand, 5’-CCGGAUGGACUAUUUCAAUTT-3’, and antisense strand, 5’-AAGUGCCAAUUACGUGUCATT-3’. siRNA transfection was conducted using Lipofectamine RNAiMAX reagent in accordance with the manufacturer’s instructions. Following transfection, the cells were lysed and subjected to Western blot analysis for subsequent evaluation.

### Cell proliferation assay

Cell proliferation was assessed using a standardized assay. Cells were cultured in 96-well plates at a density of 5,000 cells per well for a predetermined period. After a 24-hour treatment, 10 µL of CCK-8 reagent (MedChemExpress, Shanghai, China) was introduced to each well, followed by a 2-hour incubation. Subsequently, the absorbance at 450 nm was measured with a microplate reader to quantify cell proliferation.

### Wound healing assay

Cells were cultured until they reached 80–90% confluency. For the wound healing assay, confluent cells were subjected to serum starvation overnight and subsequently scraped using a sterile 200 µL pipette tip. Following a PBS wash, the cells were incubated in a medium supplemented with 2% FBS. Microscopic images (40× magnification) of the same areas were captured at the initial stage of wound formation and again at 24 h post-wound formation.

### Cell invasion assay

An invasion assay was conducted utilizing Matrigel-coated Transwell membrane inserts with 8-µm pores, as provided by Corning and BD Biosciences Durham, following the manufacturer’s instructions. In summary, after an overnight period of serum starvation, 5 × 10^^4^ cells suspended in 200 µL of serum-free medium were placed in the upper chamber. These chambers were then positioned into 24-well plates containing medium enriched with 10% bovine serum albumin. The cells were permitted to migrate for 16 h at 37 °C. Subsequently, cells that successfully traversed to the lower chamber were fixed with 4% paraformaldehyde and stained with 1% crystal violet, both reagents sourced from Solarbio.

## Flow cytometric analysis

The apoptosis of TNBC cells was assessed using flow cytometry with the Annexin V-FITC/propidium iodide (PI) Apoptosis Detection Kit (Life Technologies, Carlsbad, CA, USA). Following incubation with FITC-Annexin V and PI, the rates of apoptosis were quantified using a FACSCalibur flow cytometer (BD Biosciences, San Jose, CA, USA).

### Immunofluorescence staining

Cells under the specified conditions were fixed using 4% paraformaldehyde for 15 min at room temperature. Subsequently, they were permeabilized with 0.1% Triton-X100 in PBS and incubated in a blocking buffer at room temperature for 30 min. The cells were then incubated with a Beta Catenin Polyclonal antibody (Proteintech, Cat.51067) overnight at 4 °C, followed by three washes with PBS. Thereafter, the samples were incubated with a secondary antibody at room temperature for 1 h. The nuclei were counterstained with DAPI (5 mg/mL, Sigma). Visualization of the cells was performed using a Zeiss AxioVision imaging system (Zeiss, Oberkochen, Germany).

### Measurement of Wnt activity

TNBC cells were seeded at a density of 5,000 cells per well in white-bottomed 96-well plates. The cells underwent serum starvation overnight and were subsequently co-transfected with 0.2 µg of either TOPflash or FOPflash expression plasmids (Millipore, Temecula, CA, USA), along with 0.1 µg of the pRL-TK vector (Renilla-TK-luciferase, Promega) as a control, utilizing Lipofectamine 3000 for transfection. Firefly luciferase activity was normalized to account for transfection efficiency by dividing it by the Renilla luciferase activity. The ratio of TOPflash to FOPflash (TOP/FOP ratio) served as an indicator of β-catenin-mediated transcriptional activity. Average activity and standard deviations were derived from octopulate transfected samples.

### Western blot analysis

Cells were lysed with the highly efficient RIPA buffer (Beyotime). Following electrophoresis, proteins were transferred to a PVDF membrane and blocked using TBST buffer with 5% non-fat dry milk. The membrane was incubated overnight with the primary antibody. Following this, the protein was incubated with an HRP-conjugated secondary antibody for one hour, and visualization was accomplished using ECL chemiluminescence (MedChemExpress). The ADH1A antibody (ab108203) was obtained from Abcam (TX, USA). N-cadherin Polyclonal antibody (Cat.22018), E-cadherin Polyclonal antibody (Cat. 20874), Vimentin Polyclonal antibody (Cat. 22031), Beta Catenin Polyclonal antibody (Cat.51067), Beta actin Polyclonal antibody (Cat.66009) and HRP-labeled secondary antibodies were from Proteintech (Wuhan, China).

### Statistical analysis

A Chi-Square test was conducted to analyze the differences between the two groups. The prognostic significance, specifically overall survival (OS), was assessed using Kaplan-Meier curve analysis in conjunction with a two-tailed log-rank test. Pearson’s correlation analysis was employed to determine the degree of correlation between ADH1A and β-catenin. All statistical analyses were executed using R software (version 4.3.2), with a significance threshold set at *P* < 0.05.

## Results

### Tyrosine metabolism was correlated with prognosis in TNBC

**Fig. 1:**
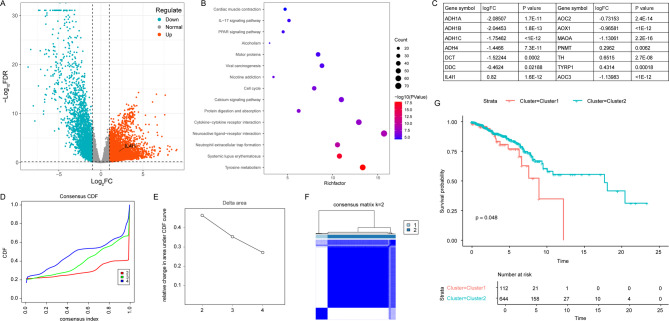
Identification of Tyrosine Metabolism Genes Influencing TNBC Prognosis. (**A**) Volcano plot depicting differentially expressed genes (DEGs) in the TCGA-TNBC cohort. (**B**) KEGG pathway enrichment analysis for both upregulated and downregulated DEGs. KEGG pathway map adapted from Kanehisa et al. (KEGG: kyoto encyclopedia of genes and genomes. Nucleic Acids Res. 2000;28(1):27–30). Kanehisa Laboratories. Used with permission. (**C**) Identification of 14 tyrosine metabolism-related genes (TRGs). LogFC (Log Fold Change) represents the expression ratio of a specific gene in tumor tissue relative to normal tissue. (**D**) Consensus clustering cumulative distribution function (CDF) for k 2 to 4. (**E**) Relative change in area under the CDF curve for k 2 to 4. (**F**) Consensus clustering matrix for k = 2. (**G**) Kaplan-Meier overall survival curves for the two clusters.

Utilizing data from the TCGA-BRCA cohort, we identified a total of 2,794 differentially expressed genes (DEGs) at the mRNA level, meeting the criteria of a false discovery rate < 0.05 and an absolute log fold change (|logFC|) ≥ 1 (Fig. 1A). Subsequent analyses using the KEGG pathway revealed that tyrosine metabolism was among the most enriched pathways associated with TNBC (Fig. 1B). A total of 14 TRGs were identified (Fig. 1C), including 10 downregulated genes (*ADH1A*, *ADH1B*, *ADH1C*, *ADH4*, *DCT*, *DDC*, *AOX1*, *MAOA*, *AOC2*, *AOC3*) and 4 upregulated genes (*IL4I1*, *PNMT*, *TH*, *TYRP1*). Based on the expression profiles of 14 prognostic tumor-related genes (TRGs), “Consensus Cluster Plus” and cumulative distribution function (CDF) analysis identified an optimal clustering number of two (designated as C1 and C2 clusters) for the TCGA-BRCA samples. We elected k = 2 as our subtype-dividing value for further study due to the similar number of samples in each cluster (Fig. 1D-F). Furthermore, Kaplan-Meier survival analysis demonstrated that the C2 cluster exhibited significantly better overall survival (OS, *p* < 0.01) compared to the C1 cluster within the TCGA datasets (Fig. 1G).

**Fig. 2:**
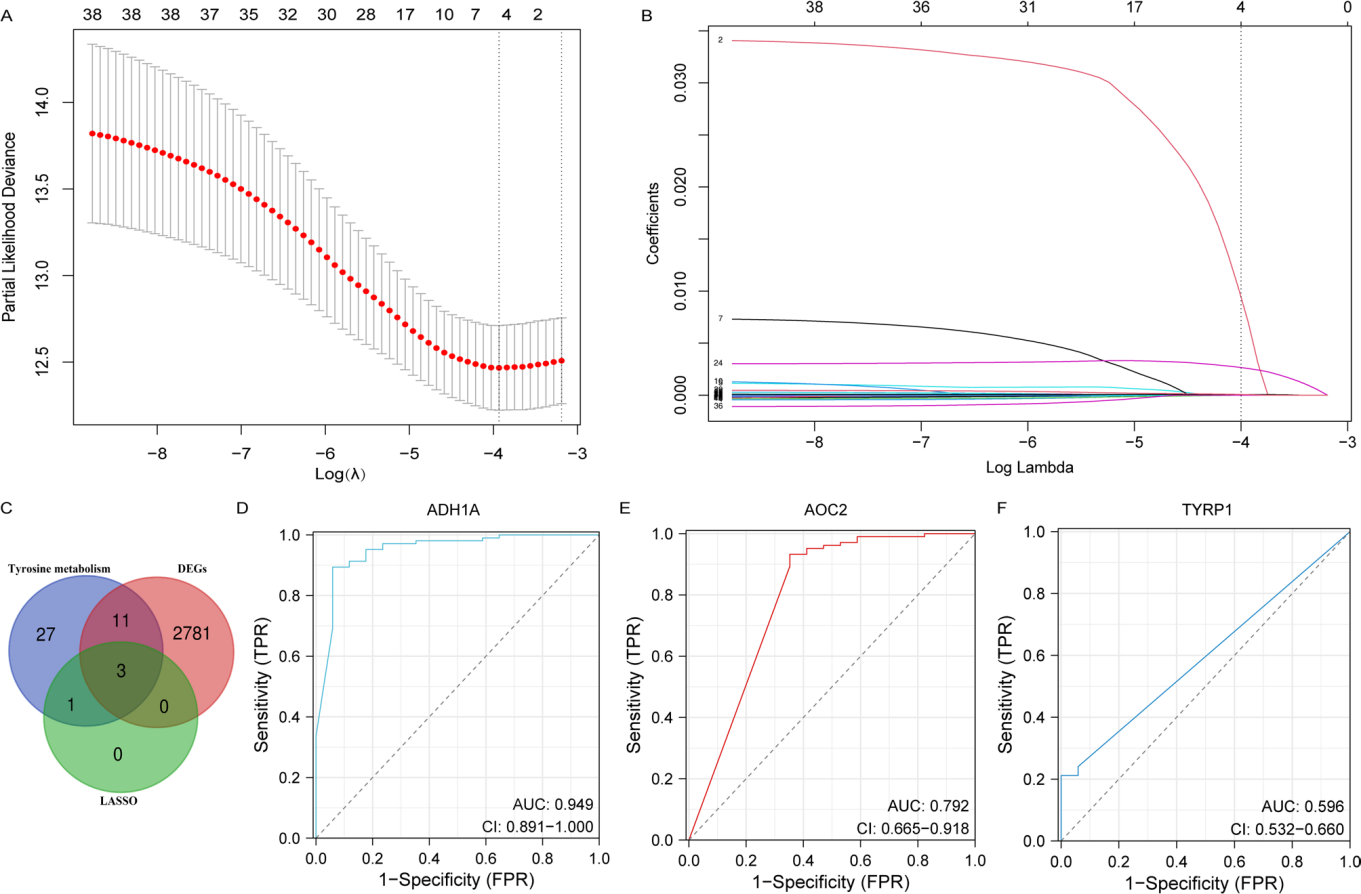
Key Tyrosine metabolism genes involved in TNBC progression. (**A**) Ten-fold cross-validation for tuning parameter selection using LASSO regression. (**B**) Coefficient screening under LASSO analysis. (**C**) Venn diagram showing the overlap of genes from differentially expressed genes (DEGs), tyrosine metabolism-related genes (TRGs), and LASSO regression analysis. (**D-F**) ROC curves demonstrating the sensitivity and specificity of 5-year overall survival (OS) predictions based on the expression levels of ADH1A (D), AOC2 (E), and TYRP1 (F).

A ten-fold cross-validation of the LASSO regression was conducted to determine the optimal lambda value based on the deviance of the least partial likelihood (Fig. 2A). The analysis uncovered a notable correlation between the lambda values and the four TRGs (Fig. 2B). A Venn diagram further identified three potential key TRGs in triple-negative breast cancer (TNBC), namely ADH1A, AOC2, and TYRP1 (Fig. 2C). However, when utilizing the Receiver Operating Characteristic (ROC) curve to predict 5-year overall survival (OS) of TNBC, ADH1A demonstrated the highest Area Under the Curve (AUC) at 0.949 (95% CI: 0.891-1.000), whereas AOC2 (AUC = 0.792) and TYRP1 (AUC = 0.596) exhibited relatively lower AUC values (Fig. 2D-F). Therefore, we further investigate the expression and biological function of ADH1A in TNBC.

### ADH1A is downregulated in patients with TNBC associated with poor prognosis

**Fig. 3:**
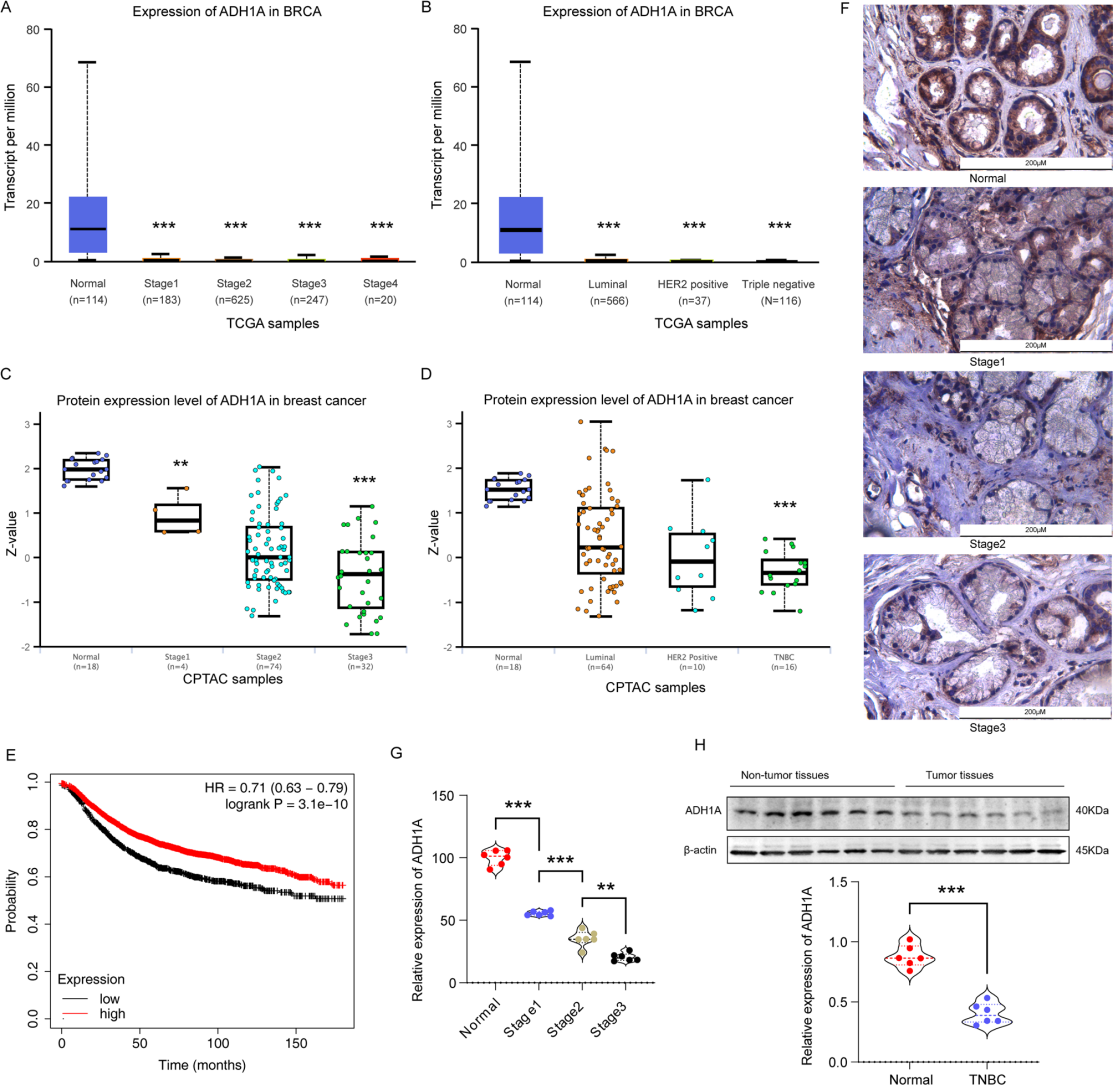
The low expression of ADH1A and its association with a worse prognosis in TNBC. (**A**) Significant reduction of ADH1A mRNA levels in tumor tissues across different stages, as observed in the TCGA database. (**B**) Marked decrease in ADH1A mRNA levels in various types of breast cancer. (**C**) Protein levels of ADH1A are lower in tumor tissues at different stages, as indicated by the CPTAC database. (**D**) ADH1A protein levels in tumor tissues differ across various breast cancer types based on CPTAC data. (E) Kaplan-Meier survival analysis from the TCGA dataset demonstrates the prognostic capacity of ADH1A in TNBC. (**F-G**) Representative immunohistochemistry images and quantitative assessment of ADH1A expression in TNBC tissues at different stages (Bar = 200 μm). (**H**) Western blot analysis and quantitative assessment of ADH1A protein levels in six TNBC tissues compared to adjacent normal tissues. **P < 0.01, ***P < 0.001.

Analysis of the TCGA-BRCA cohort data indicates a significant reduction in ADH1A mRNA expression levels across breast cancers with varying degrees of invasiveness (Fig. 3A), with the most pronounced decrease observed in TNBC (Fig. 3B). Complementary findings from the CPTAC database corroborate this trend, demonstrating a similar reduction in ADH1A protein expression in both high-grade breast cancer (Fig. 3C) and TNBC (Fig. 3D). Further investigation using the Kaplan–Meier method revealed a significant correlation between decreased ADH1A expression and overall survival (OS) in TNBC patients, with a hazard ratio (HR) of 0.71 and a P-value of less than 0.001 (Fig. 3E). Given the limited cohort size of our TNBC samples, we extended ADH1A expression analysis to TNBC tissues from three GEO datasets (GSE38959, GSE45827, GSE65194). Heatmaps visualizing differentially expressed genes between TNBC and normal tissues (Supplementary Fig. 1A-C) and comparative boxplots demonstrating consistent ADH1A downregulation (Supplementary Fig. 1D-F) are presented. To elucidate the impact of ADH1A on TNBC, we conducted a study involving six TNBC patients. Employing immunohistochemistry and Western blot techniques, we confirmed a marked decrease in ADH1A expression in TNBC tissues (Fig. 3F-H). Building on above results, we proceeded with subsequent cellular functional assays.

### ADH1A regulated malignant behavior of TNBC cells

**Fig. 4:**
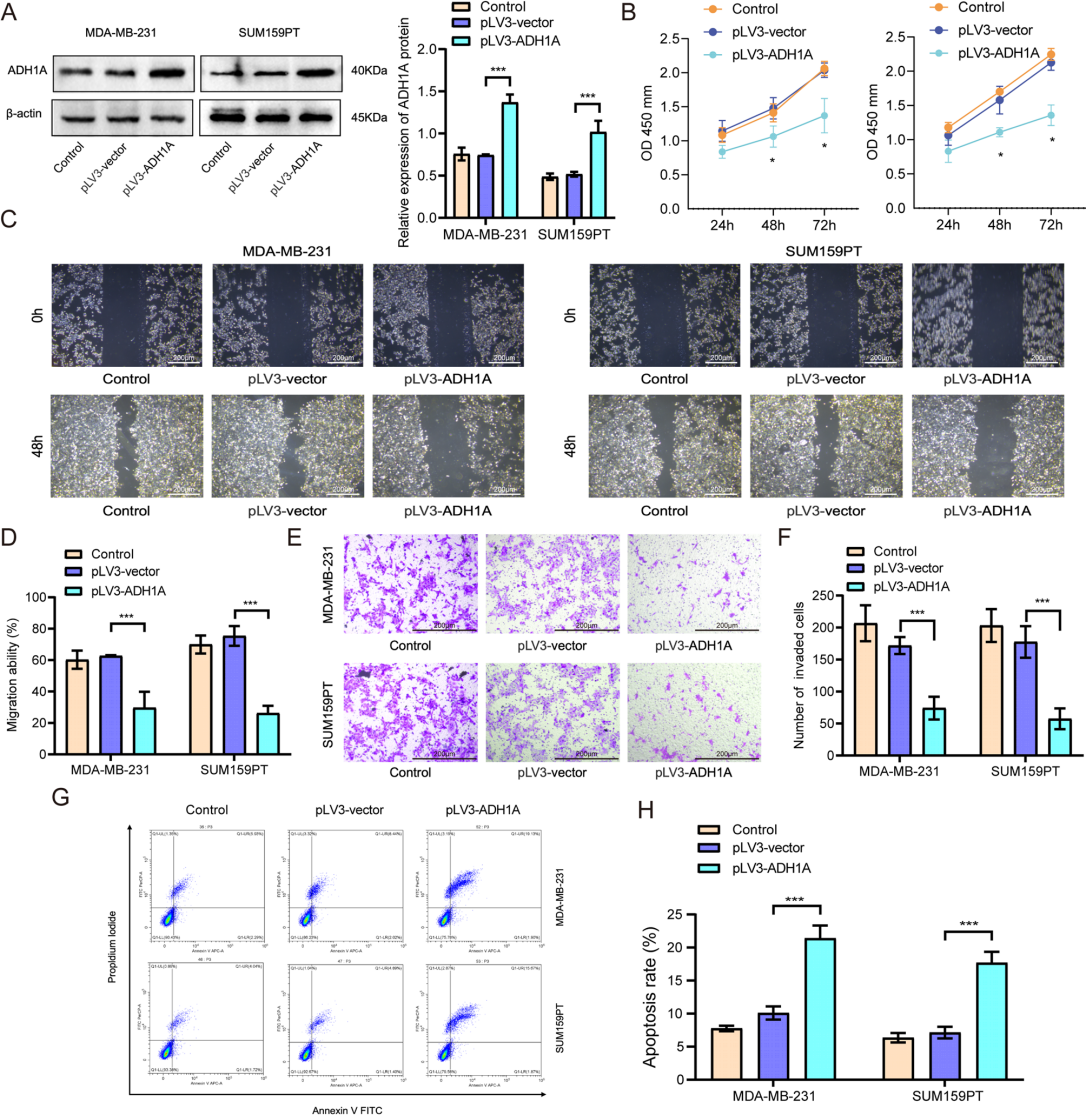
Knockdown of ADH1A promoted cell proliferation and invasion of TNBC cells. (**A**) Western blot analysis confirmed the knockdown of ADH1A in MDA-MB-231 and SUM159PT cells. (**B**) CCK-8 assay results showed that ADH1A silencing suppressed cell proliferation in MDA-MB-231 and SUM159PT cells. (**C-D**) Wound healing assays demonstrated that knockdown of ADH1A enhanced cell migration. (**E-F**) Transwell invasion assays revealed that knockdown of ADH1A increased the invasion capability of TNBC cells. (**G-H**) Flow cytometry analysis indicated the influence of ADH1A silencing on cell apoptosis in TNBC cells. *P < 0.05, **P < 0.01, ***P < 0.001.

**Fig. 5:**
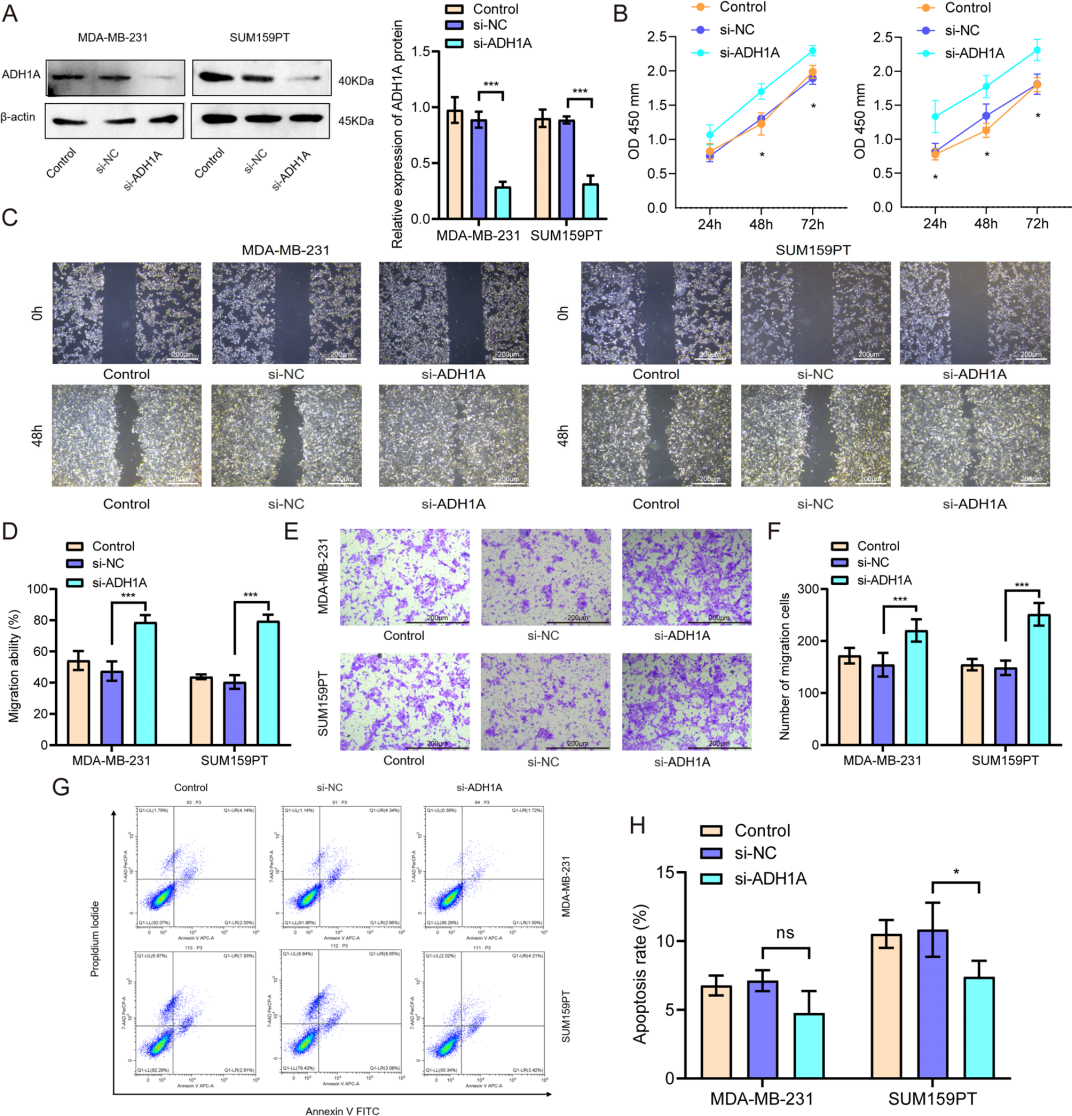
Overexpression of ADH1A inhibited cell proliferation, invasion and promotes apoptosis of TNBC cells. (**A**) Western blot analysis confirmed the overexpression of ADH1A in MDA-MB-231 and SUM159PT cells. (**B**) CCK-8 assay results showed that ADH1A overexpression inhibited cell proliferation in MDA-MB-231 and SUM159PT cells. (**C-D**) Wound healing assays demonstrated that ADH1A overexpression significantly reduced the migration of TNBC cells. (**E-F**) Transwell invasion assays revealed that ADH1A overexpression decreased the invasion capability of TNBC cells. (**G-H**) Flow cytometry analysis indicated that ADH1A overexpression promoted apoptosis in TNBC cells. **P < 0.01, ***P < 0.001.

In MDA-MB-231 and SUM159PT cells, we used specific siRNA to knockdown of ADH1A (Fig. 4A). As expected, knockdown of ADH1A promoted cell proliferation of both MDA-MB-231 and SUM159PT cells (Fig. 4B). In addition, the wound-healing assay and cell invasion assays corroborated that knockdown of ADH1A enhanced cell migration (Fig. 4C-D) and invasion (Fig. 4E-F) in above two cell lines. Subsequent flow cytometry analysis revealed that knockdown of ADH1A suppressed cell apoptosis in SUM159PT cells, but not in MDA-MB-231 cells (Fig. 4G-H). One the contrary, following the transfection of TNBC cell lines MDA-MB-231 and SUM159PT with pLV3-ADH1A, a significantly increased expression of the ADH1A protein compare with the cells transfected with the empty vector (pLV3-vector) (Fig. 5A). The overexpression of ADH1A was associated with a reduction in cell proliferation in TNBC cells (Fig. 5B). Furthermore, upregulation of ADH1A significantly impeded cell migration in both MDA-MB-231 and SUM159PT cell lines (Fig. 5C-D). Consistently, ADH1A overexpression substantially inhibited the invasive capacity of these cells (Fig. 5E-F). Overexpression of ADH1A also significantly enhanced apoptosis of MDA-MB-231 and SUM159PT cells (Fig. 5G-H). Collectively, these findings suggest that ADH1A controls the malignant behavior of TNBC cells, in vitro.

### ADH1A affected TNBC cell epithelial mesenchymal transition (EMT) via the Wnt/β-catenin pathway

**Fig. 6:**
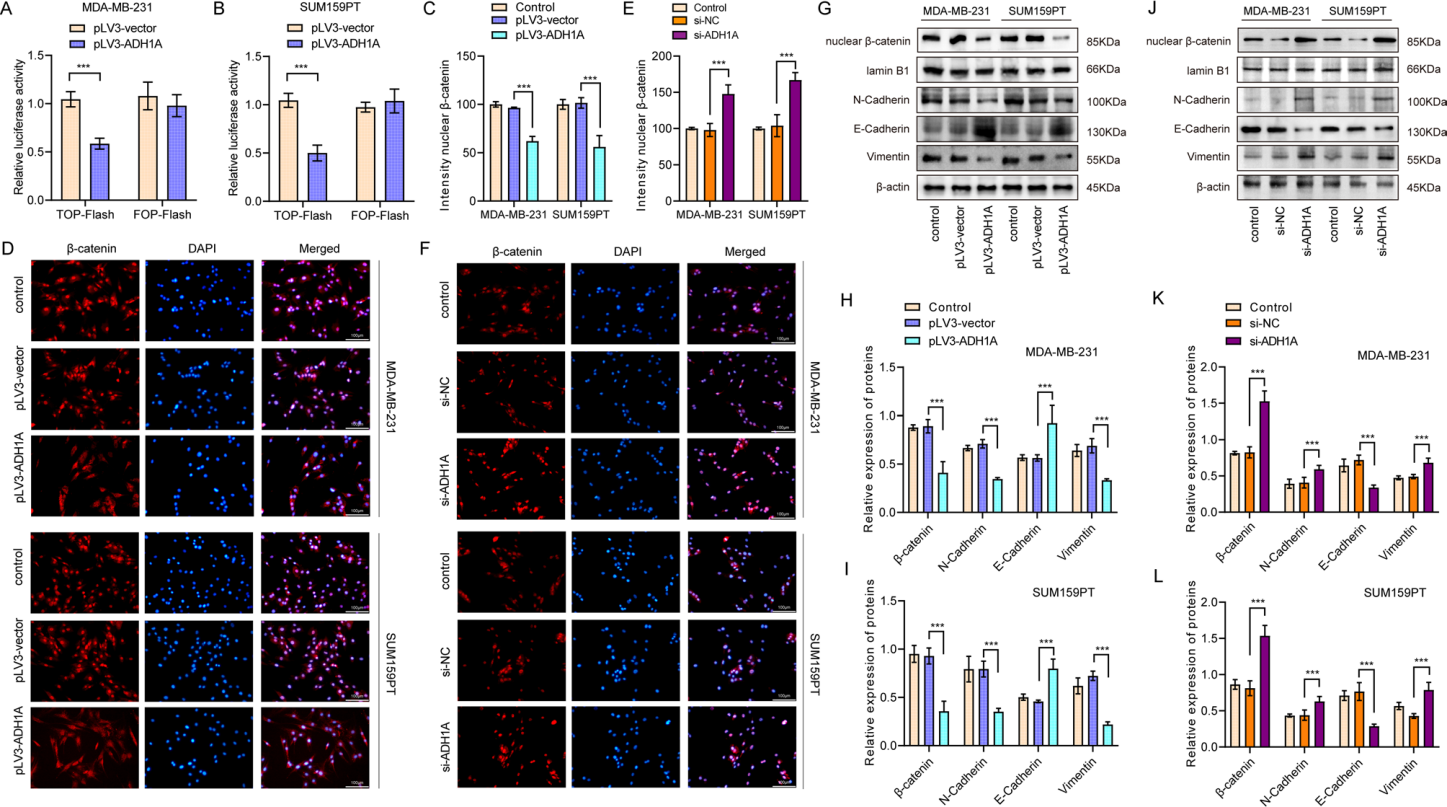
ADH1A regulated epithelial mesenchymal transition (EMT) of TNBC cells through Wnt/β-catenin signaling. (**A-B**) The TOP/FOP assay was conducted to assess Wnt signaling pathway activity in cells with and without ADH1A overexpression. (**C-D**) Immunofluorescence assay demonstrated the nuclear translocation of β-catenin following the indicated treatment, with cell nuclei stained using DAPI (blue), in TNBC cells with overexpression of ADH1A. (**E-F**) Immunofluorescence assay showed the fluorescence intensity of nuclear β-catenin was increased after knockdown of ADH1A. (**G-I**) Western blot analysis and quantitative assessment showed the levels of β-catenin and EMT markers E-cadherin, N-cadherin, and Vimentin in both ADH1A overexpression and control groups in MDA-MB-231 and SUM159PT cells. (**J-L**) Western blot analysis and quantitative assessment showed the levels of β-catenin and EMT markers in both ADH1A silencing and control groups in MDA-MB-231 and SUM159PT cells. **P < 0.01, ***P < 0.001.

Given the role of the Wnt/β-catenin signaling pathway in mediating EMT and promoting the malignant characteristics of TNBC, as well as its potential as a therapeutic target^12^, we conducted an investigation into the impact of ADH1A on EMT and β-catenin. Notably, nuclear accumulation of β-catenin is indicative of Wnt/β-catenin pathway activation. We firstly detected the expression change of EMT markers in our clinical cohorts and observed that, increased TNBC tumor grade correlated with elevated expression of β-catenin and N-cadherin, alongside decreased E-cadherin expression (Supplementary Fig. 2A-D). Our analyses further revealed an inverse correlation between ADH1A and β-catenin expression in TCGA TNBC cohorts (Supplementary Fig. 2E). In the GSE76124 dataset, ADH1A demonstrated positive correlation with *CDH1* (E-cadherin), negative correlations with *CDH2* (N-cadherin) and *VIM* (vimentin) (Supplementary Fig. 2F-H). To assess β-catenin activity, we employed TOP/FOP luciferase reporter assays, which demonstrated that ADH1A overexpression resulted in a reduction of TOP/FOP luciferase activity in both MDA-MB-231 and SUM159PT cell lines (Fig. 6A-B). Subsequent immunofluorescence assays demonstrated that overexpression of ADH1A resulted in a reduction of nuclear β-catenin levels compared to the control group (Fig. 6C-D). Contrarily, knockdown of ADH1A increased nuclear β-catenin expression (Fig. 6E-F). Additionally, ADH1A overexpression was associated with decreased protein expression of β-catenin and epithelial-mesenchymal transition (EMT) markers, including N-cadherin, E-cadherin, and Vimentin (Fig. 6G-I). While ADH1A silencing facilitated the EMT of BC cells, as evidenced by increased N-cadherin, E-cadherin, and Vimentin protein expressions (Fig. 6J-L).

**Fig. 7:**
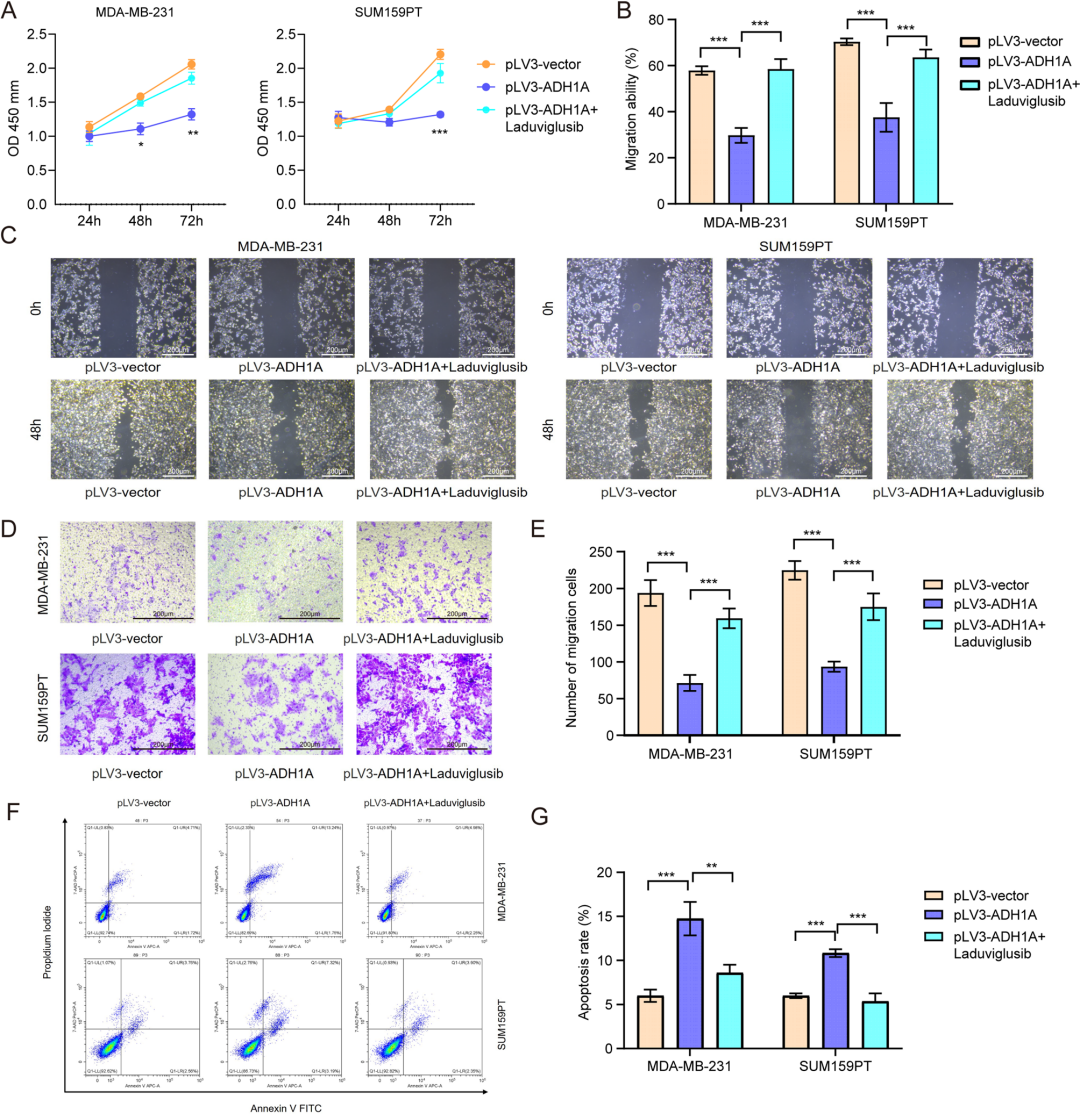
Reactivation of Wnt/β-catenin signaling reverses ADH1A mediated tumor suppression in TNBC cells. (**A**) CCK-8 proliferation assay in ADH1A-overexpressing MDA-MB-231 and SUM159PT cells ± Wnt agonist Laduviglusib (5 µM). (**B-C**) Wound healing assays demonstrating Laduviglusib-mediated rescue of ADH1A-inhibited migration. (**D-E**) Transwell invasion assays showing abolition of ADH1A-suppressed invasion by Laduviglusib. (**F-G**) Flow cytometry quantification of Laduviglusib-induced apoptosis attenuation in ADH1A-overexpressing cells. Data presented as mean ± SD; **P < 0.01, ***P < 0.001 versus ADH1A-OE control (without Laduviglusib).

To further validate the role of Wnt/β-catenin signaling pathway in ADH1A’s role in regulating TNBC, we treated cells with a canonical Wnt/β-catenin signalling pathway activator (Laduviglusib) to assess if activating Wnt/β-catenin rescues the inhibitory effects of ADH1A overexpression on cell proliferation, migration and invasion. The results in Fig. 7A-E showed that activation of Wnt signaling counteracted the tumor-suppressive effects of ADH1A overexpression, restoring cell proliferation, migration and invasion closer to control levels. Furthermore, addition with Laduviglusib also significantly suppressed apoptosis in ADH1A overexpressed MDA-MB-231 and SUM159PT cells (Fig. 7F-G). These findings suggest that ADH1A may inhibit EMT in TNBC cells via modulation of the Wnt/β-catenin signaling pathway.

## Discussion

Tyrosine metabolism is crucial in substance catabolism, and its dysregulation has been identified as a significant marker in disease progression and tumorigenesis^13^. Numerous reports have demonstrated that genes linked to the tyrosine metabolism pathway contribute to the progression of prostate cancer and may act as prognostic indicators for cancer patients^4,14^. Additionally, emerging research indicates a possible relationship between tyrosine metabolism and tumor immunogenicity^15,16^. Despite significant advancements in the diagnosis, treatment, and prognostication of breast cancer, the understanding of the potential mechanisms driving the onset and progression of TNBC remains rudimentary. Breast carcinoma, originating from the epithelial tissue of the breast, ranks among the most prevalent malignant tumors^17^. Nevertheless, the comprehensive impact and association between gene signatures and tumor proliferation, mediated by multiple TRGs in TNBC, have yet to be fully elucidated^18^.

Tyrosine metabolism is facilitated by a series of enzymatic reactions, and the absence of activity in any of these enzymes can result in the accumulation of toxic metabolites, which may cause damage to the liver, kidneys, nervous system, or other organs^19,20^. The tyrosine metabolism pathway has been observed to be inhibited in hepatocellular carcinoma^21^. Through bioinformatics analysis, we were the first to identify that tyrosine metabolism is also closely associated with TNBC in TCGA cohorts, and it is negatively correlated with patient prognosis. To elucidate the role and mechanism of tyrosine-metabolizing enzymes in triple-negative breast cancer (TNBC), we employed machine learning techniques to identify key proteins involved in tyrosine metabolism during the progression of TNBC. Our findings indicate that only ADH1A holds potential as a diagnostic marker for TNBC. Furthermore, prognostic gene analysis categorized TNBC patients into two subtypes (C1 and C2). Notably, the C2 cluster, associated with a poorer prognosis, exhibited lower expression levels of several oxidases and dehydrogenases, including *ADH1 (A*,* B*,* C)*,* ADH4*,* AOX1*,* IL4I1*, and *MAOA*. The findings indicate the potential efficacy of antioxidant therapy in impeding the progression of TNBC^22^. Additionally, ADH1A is integral to various metabolic processes, including the catabolism of norepinephrine, dopamine, serotonin, and bile acids^23^.

The human *ADH1A*, *ADH1B*, and *ADH1C* genes encode alcohol dehydrogenases (ADHs) responsible for the metabolism of ethanol. These genes have evolved through recent tandem duplications and share similar proximal cis-acting elements, yet they exhibit differences in tissue-specific expression^24^. In addition to their primary role in ethanol metabolism, the recombinant ADH1 protein demonstrates further biological functions. Intracellularly, ADH1 plays a critical role in regulating the redox state of substrates, thereby influencing cellular metabolism and growth^25^. Elevated expression of ADH1A was associated with favorable survival outcomes in patients with hepatocellular carcinoma^26^. A recent study demonstrated that ADH1A overexpression inhibits cell proliferation, migration, and invasion of gastric cancer, while facilitating cell apoptosis and the secretion of inflammatory factors^27^. In our study, we have, for the first time, demonstrated that ADH1A is significantly downregulated in TNBC. Furthermore, overexpression of ADH1A was found to suppress cell proliferation, migration, and invasion, while promoting apoptosis in TNBC cells. Moreover, our findings suggest that these effects may be attributed to the inhibition of the Wnt/β-catenin signaling pathway. The Wnt/β-catenin pathway is a critical developmental signaling cascade that plays a significant role in oncogenesis^28^. This pathway involves the nuclear translocation of β-catenin, which activates the expression of Wnt target genes through interactions with various transcription factors. Importantly, Wnt signaling is recognized for its regulation of several cellular functions, including the modulation of proliferation^29^, facilitation of DNA damage repair, and inhibition of apoptosis^30^. Here, we found that knockdown of AHD1A promotes the malignant behavior of TNBC cells, whereas AHD1A overexpression leading to the inhibition of nuclear β-catenin, thus to confer anticancer property in TNBC, indicating AHD1A as a potential cancer suppressor gene.

The present study is subject to certain limitations. Primarily, our investigation into the mechanism by which ADH1A influences the function of TNBC cells was confined at cellular level. While this study elucidates ADH1A’s tumor-suppressive mechanism at the cellular level, we acknowledge that validation in in vivo models is warranted to fully characterize its therapeutic potential. Constraints in animal facility capabilities for handling genetically modified TNBC cell lines currently preclude such experiments. Future studies utilizing patient-derived xenografts or alternative models are planned to address this gap. However, the inverse correlation between ADH1A and mesenchymal markers in TNBC cohort clinically mirrors our in vitro findings. This real-world evidence positions ADH1A as a gatekeeper of epithelial phenotype, potentially through β-catenin sequestration. It is noteworthy that genetic variation in ADH1A may lead to phenotypic diversity, including individual genetic variability in susceptibility to substance dependence^31^. Nevertheless, our study focused exclusively on the impact of ADH1A expression regulation on breast cancer cells, leaving its variation and mutation status in TNBC inadequately understood. Further population-based research is required to elucidate the function of ADH1A in various cancers.

## Conclusion

In sum, this study explored TCGA-BRAC databases using bioinformatics methods, identifying TRGs that affect TNBC progression. Decreased AHD1A correlates with poor prognosis in TNBC. In vitro, ADH1A was found to suppress the malignant progression of TNBC through regulating the Wnt/β-catenin signaling pathway. Our findings provide a novel therapeutic target for TNBC.

## Supplementary Information

Below is the link to the electronic supplementary material.

Supplementary Material 1

Supplementary Material 2

## Data Availability

All data generated or analysed during this study are included in this published article.

## References

[1] Arnold, M. et al. Current and future burden of breast cancer: global statistics for 2020 and 2040 [J]. *Breast* **66**, 15–23 (2022). 36084384 10.1016/j.breast.2022.08.010 PMC9465273

[2] Harbeck, N. et al. Breast cancer [J]. *Nat. Reviews Disease Primers*. **5** (1), 66 (2019). 31548545 10.1038/s41572-019-0111-2

[3] Vagia, E., Mahalingam, D. & Cristofanilli, M. The landscape of targeted therapies in TNBC [J]. *Cancers (Basel) ***12**(4), 916 (2020). 10.3390/cancers12040916 PMC7226210 32276534

[4] Wiggins, T. et al. Tyrosine, phenylalanine, and Tryptophan in gastroesophageal malignancy: a systematic review [J]. *Cancer Epidemiol. Biomarkers Prev.* **24** (1), 32–38 (2015). 25344892 10.1158/1055-9965.EPI-14-0980

[5] Lai, H. S. et al. Plasma free amino acid profile in cancer patients [J]. *Semin Cancer Biol.* **15** (4), 267–276 (2005). 15894488 10.1016/j.semcancer.2005.04.003

[6] Zhou, Y. et al. Identification and validation of a tyrosine metabolism-related prognostic prediction model and characterization of the tumor microenvironment infiltration in hepatocellular carcinoma [J]. *Front. Immunol.* **13**, 994259 (2022). 36341373 10.3389/fimmu.2022.994259 PMC9633179

[7] Tian, W. et al. Novel implication of the basement membrane for breast cancer outcome and immune infiltration [J]. *Int. J. Biol. Sci.* **19** (5), 1645–1663 (2023). 37056938 10.7150/ijbs.81939 PMC10086744

[8] Tian, W. et al. Autophagy deficiency induced by SAT1 potentiates tumor progression in Triple-Negative breast cancer [J]. *Adv Sci (Weinh).* **11 **(36), e2309903 (2024). 10.1002/advs.202309903 PMC11423137 39073262

[9] Kanehisa, M. et al. KEGG as a reference resource for gene and protein annotation [J]. *Nucleic Acids Res.* **44** (D1), D457–D462 (2016). 26476454 10.1093/nar/gkv1070 PMC4702792

[10] Kanehisa, M. & Goto, S. KEGG: Kyoto encyclopedia of genes and genomes [J]. *Nucleic Acids Res.* **28** (1), 27–30 (2000). 10592173 10.1093/nar/28.1.27 PMC102409

[11] Marastoni, S. et al. EMILIN2 down-modulates the Wnt signalling pathway and suppresses breast cancer cell growth and migration [J]. *J. Pathol.* **232** (4), 391–404 (2014). 24374807 10.1002/path.4316

[12] Yin, P. et al. Wnt signaling in human and mouse breast cancer: focusing on Wnt ligands, receptors and antagonists [J]. *Cancer Sci.* **109** (11), 3368–3375 (2018). 30137666 10.1111/cas.13771 PMC6215866

[13] Blackburn, P. R. et al. Silent tyrosinemia type I without elevated tyrosine or succinylacetone associated with liver cirrhosis and hepatocellular carcinoma [J]. *Hum. Mutat.* **37** (10), 1097–1105 (2016). 27397503 10.1002/humu.23047 PMC5108417

[14] Nguyen, T. N., Nguyen, H. Q. & Le, D. H. Unveiling prognostics biomarkers of tyrosine metabolism reprogramming in liver cancer by cross-platform gene expression analyses [J]. *PloS One*. **15** (6), e0229276 (2020). 32542016 10.1371/journal.pone.0229276 PMC7295234

[15] De Matteis, S. et al. Aberrant Metabolism in Hepatocellular Carcinoma Provides Diagnostic and Therapeutic Opportunities [J]. *Oxidative medicine and cellular longevity* **2018**, 7512159 (2018). 10.1155/2018/7512159 PMC6247426 30524660

[16] Li, W. et al. Gene Expression Analysis Reveals Prognostic Biomarkers of the Tyrosine Metabolism Reprogramming Pathway for Prostate Cancer [J]. *J. Oncol.* **2022**, 5504173 (2022). 10.1155/2022/5504173 PMC9279037 35847355

[17] Karim, A. M. et al. Triple-negative breast cancer: epidemiology, molecular mechanisms, and modern vaccine-based treatment strategies [J]. *Biochem. Pharmacol.* **212**, 115545 (2023). 37044296 10.1016/j.bcp.2023.115545

[18] Li, Y. et al. Recent advances in therapeutic strategies for triple-negative breast cancer [J]. *J. Hematol. Oncol.* **15** (1), 121 (2022). 36038913 10.1186/s13045-022-01341-0 PMC9422136

[19] Bouyacoub, Y. et al. Novel and recurrent mutations in the TAT gene in Tunisian families affected with Richner-Hanhart syndrome [J]. *Gene* **529** (1), 45–49 (2013). 23954227 10.1016/j.gene.2013.07.066

[20] Li, L. et al. Fumarylacetoacetate hydrolase Knock-out rabbit model for hereditary tyrosinemia type 1 [J]. *J. Biol. Chem.* **292** (11), 4755–4763 (2017). 28053091 10.1074/jbc.M116.764787 PMC5377789

[21] Wang, J. et al. Decreased SLC27A5 suppresses lipid synthesis and tyrosine metabolism to activate the cell cycle in hepatocellular carcinoma [J]. *Biomedicines.* **10**(2),234 (2022). 10.3390/biomedicines10020234 PMC8869743 35203444

[22] Liou, G. Y. & Storz, P. Reactive oxygen species in cancer [J]. *Free Radic Res.* **44** (5), 479–496 (2010). 20370557 10.3109/10715761003667554 PMC3880197

[23] Höög, J. O. et al. Mammalian alcohol dehydrogenase - functional and structural implications [J]. *J. Biomed. Sci.* **8** (1), 71–76 (2001). 11173978 10.1007/BF02255973

[24] Zahid, K. R. et al. mTOR/HDAC1 crosstalk mediated suppression of ADH1A and ALDH2 links alcohol metabolism to hepatocellular carcinoma onset and progression in Silico [J]. *Front. Oncol.* **9**, 1000 (2019). 31637215 10.3389/fonc.2019.01000 PMC6787164

[25] Gao, Q. et al. Integrated proteogenomic characterization of HBV-Related hepatocellular carcinoma [J]. *Cell.* **179**(2), 561 – 577 (2019). 10.1016/j.cell.2019.08.052 31585088

[26] Liu, X. et al. Prognostic implications of alcohol dehydrogenases in hepatocellular carcinoma [J]. *BMC Cancer*. **20** (1), 1204 (2020). 33287761 10.1186/s12885-020-07689-1 PMC7720489

[27] Ma, J. et al. Inflammation-Related gene ADH1A regulates the polarization of macrophage M1 and influences the malignant progression of gastric cancer [J]. *J. Inflamm. Res.* **17**, 4647–4665 (2024). 39045532 10.2147/JIR.S452670 PMC11264289

[28] Zhan, T., Rindtorff, N. & boutros, M. Wnt signaling in cancer [J]. *Oncogene.* **36** (11), 1461–1473 (2017). 27617575 10.1038/onc.2016.304 PMC5357762

[29] Niehrs, C. & Acebron, S. P. Mitotic and mitogenic Wnt signalling [J]. *Embo J.* **31** (12), 2705–2713 (2012). 22617425 10.1038/emboj.2012.124 PMC3380213

[30] De Abreu, W. A. et al. Wnt/β-Catenin Inhibition disrupts carboplatin resistance in isogenic models of Triple-Negative breast cancer [J]. *Front. Oncol.* **11**, 705384 (2021). 34367990 10.3389/fonc.2021.705384 PMC8340846

[31] Zuo, L. et al. ADH1A variation predisposes to personality traits and substance dependence [J]. *Am. J. Med. Genet. B Neuropsychiatr Genet.* **153b** (2), 376–386 (2010). 19526455 10.1002/ajmg.b.30990 PMC2861415

