# Supplementary material for: Overexpression of alcohol dehydrogenase 1 A inhibits the progress of triple negative breast cancer via Wnt/β-catenin signaling
Source: Sci Rep. 2025 Sep 26;15:32986. doi: 10.1038/s41598-025-17643-5 (PMC12475006; doi:10.1038/s41598-025-17643-5)
Supplement: Supplementary file 1 — Supplementary Material 1 [file 41598_2025_17643_MOESM1_ESM.docx]

**Supplemental Materials**

Overexpression of tyrosine metabolism-related gene ADH1A inhibits the progress of triple‐negative breast cancer via Wnt/β‐catenin signaling

**Authors**

Lihong Su, Chunlin Qiao, Jin Luo, Bianling Zhu, Yunxiao Liu*

**Institutions**

Department of Pathology, Shanxi Provincial People's Hospital, Taiyuan, 030000, China.

*Corresponding authors: Yunxiao Liu, Department of Pathology, Shanxi Provincial People's Hospital, No. 29, Shuangtasi Street, Taiyuan, 030000, Shanxi, China. Email adress: LLLyunxiao@126.com.

**Supplemental Figures**


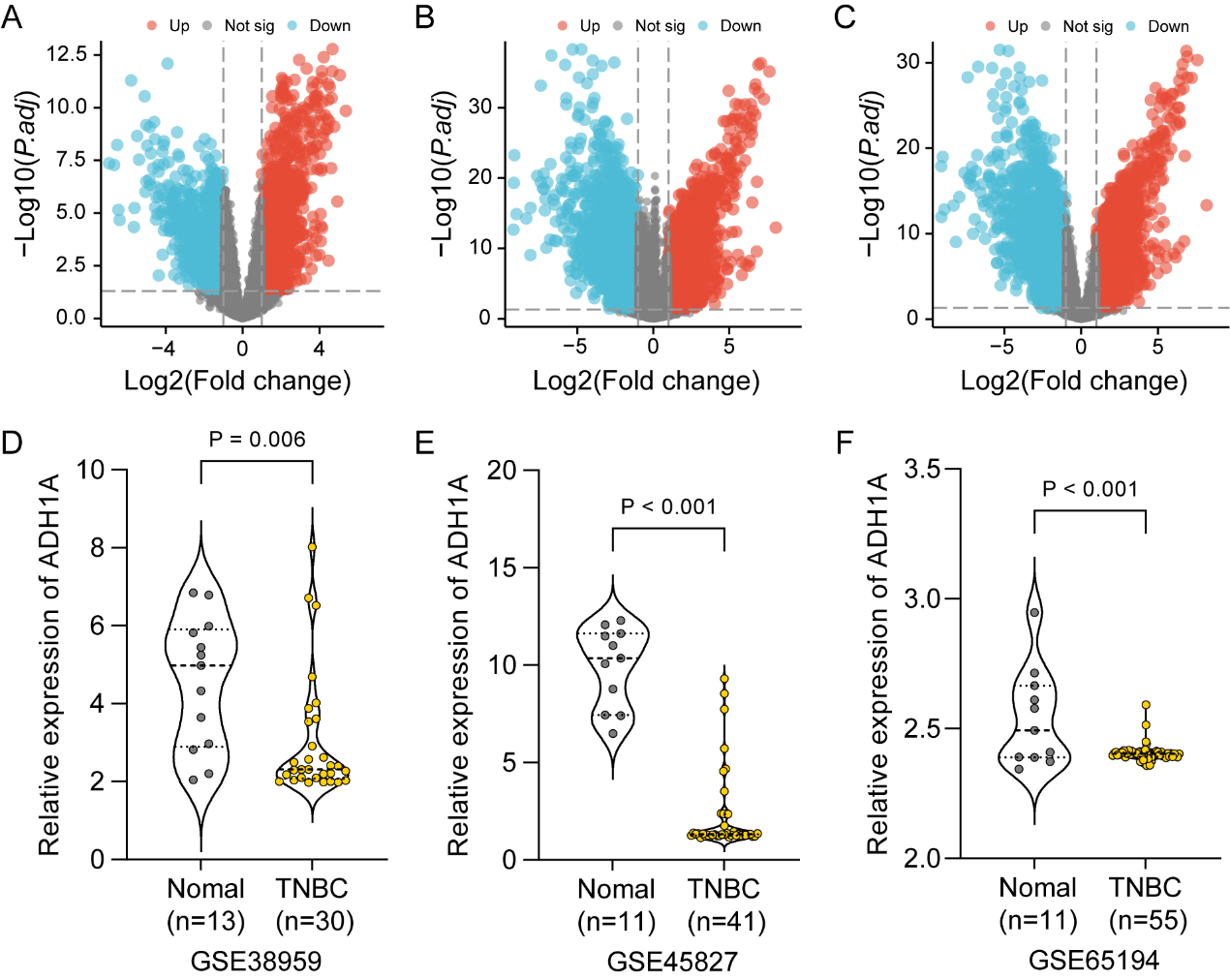


Supplementary Figure 1. ADH1A expression was decreased in TNBC samples from Gene Expression Omnibus (GEO) databases. RNA-seq data from normal breast tissues and TNBC samples were obtained from three GEO databases (GSE38959, GSE45827 and GSE65194) and the heatmap (A-C) showed differentially expressed genes between the two groups. (D-F) The boxplots showed relative expression of ADH1A gene between the two groups.


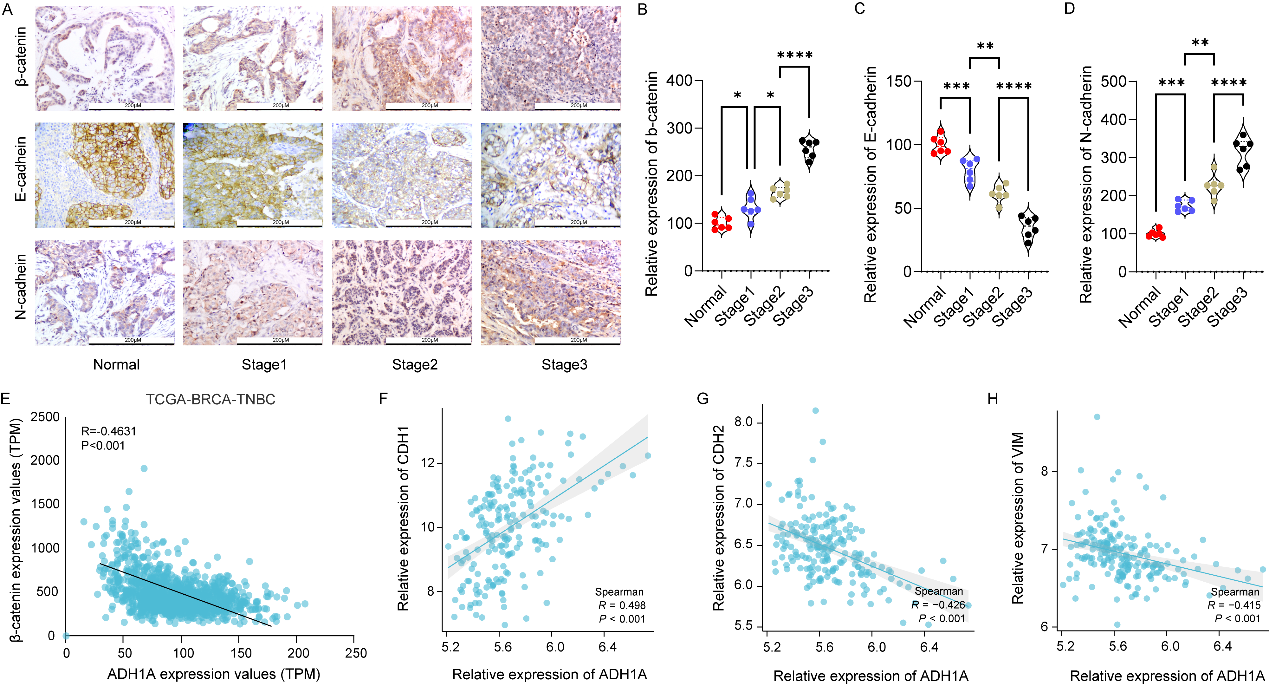


Supplementary Figure 2. ADH1A was correlated with markers of epithelial‐mesenchymal transition (EMT) in TNBC tissues. (A) The expression of EMT markers β-catenin, E-cadherin and N-cadherin in TNBC tissues of different grades was detected by immunohistochemistry (B-D) Quantitative analysis of the results from A. (E) Correlation analysis of ADH1A and β-catenin genes in TNBC tissue samples in the TCGA database. (F-G) Correlation analysis of ADH1A and EMT markers *CDH1* (E-cadherin), *CDH2* (N-cadherin) and *VIM* genes in TNBC tissue samples in the GEO database (GSE76124). *P < 0.05, **P < 0.01, ***P < 0.001.
