# Supplementary material for: Overexpression of alcohol dehydrogenase 1 A inhibits the progress of triple negative breast cancer via Wnt/β-catenin signaling
Source: Sci Rep. 2025 Sep 26;15:32986. doi: 10.1038/s41598-025-17643-5 (PMC12475006; doi:10.1038/s41598-025-17643-5)
Supplement: Supplementary file 2 — Supplementary Material 2 [file 41598_2025_17643_MOESM2_ESM.pdf]

Fig. 3

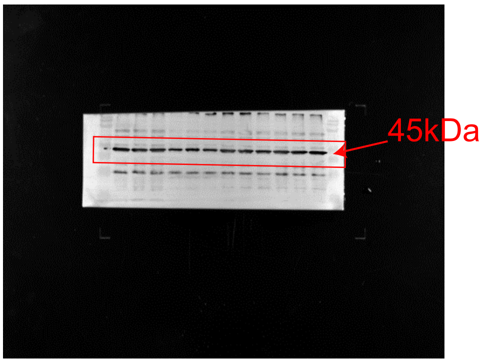

Actin

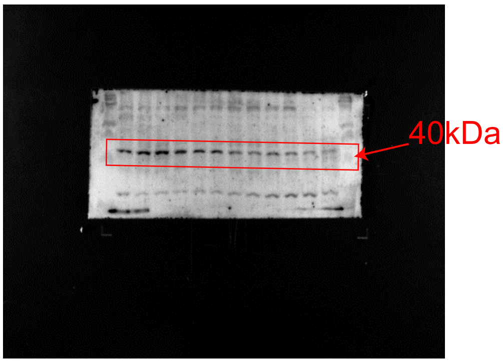

ADH1A

Fig. 4

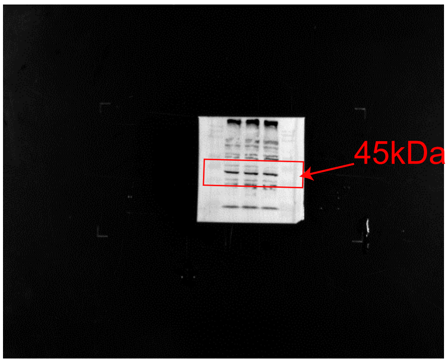

Actin

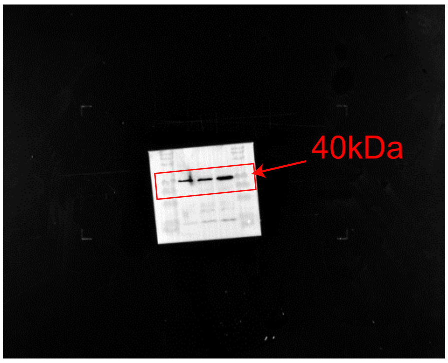

ADH1A

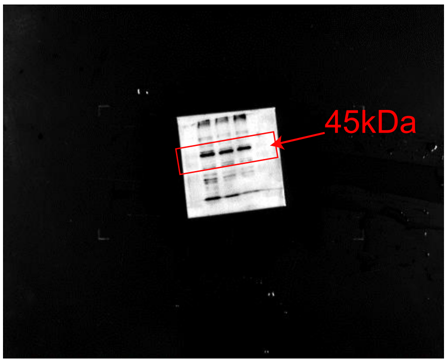

Actin

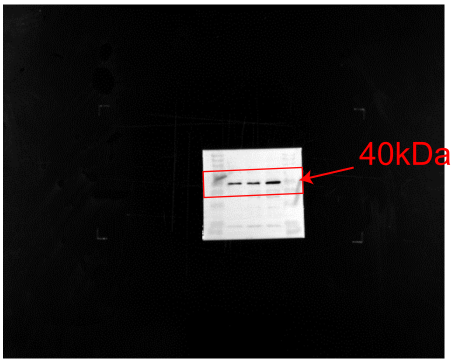

ADH1A

Fig. 5

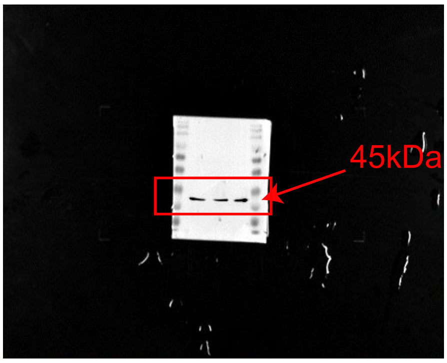

Actin

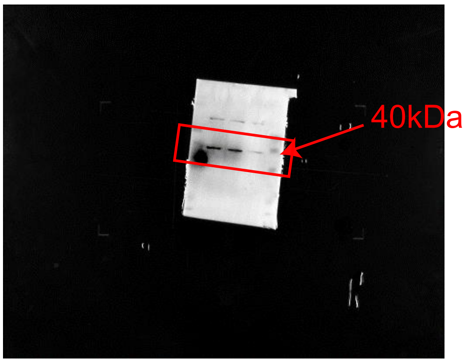

ADH1A

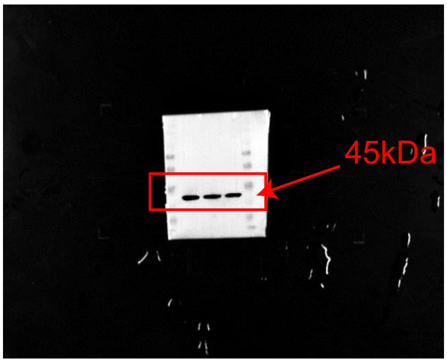

Actin

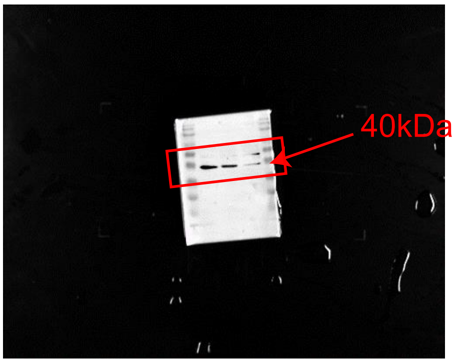

ADH1A

Fig. 6G

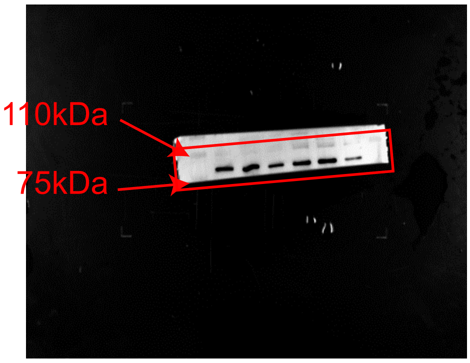

β-catenin

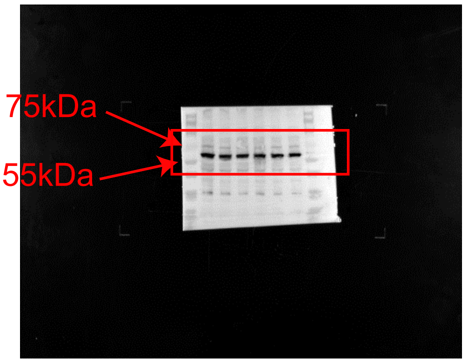

Lamin B1

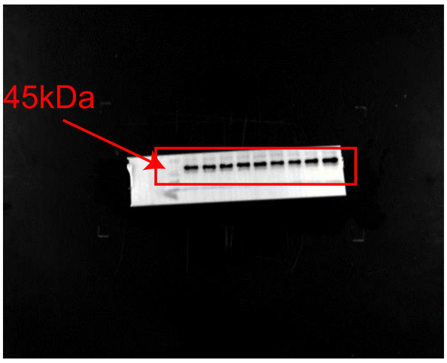

Actin

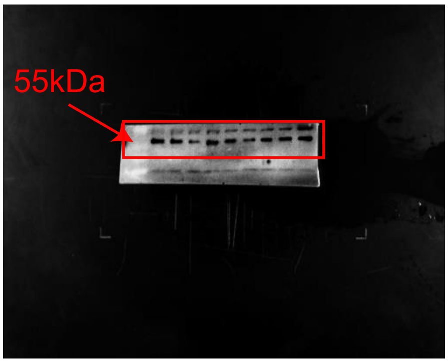

Vim

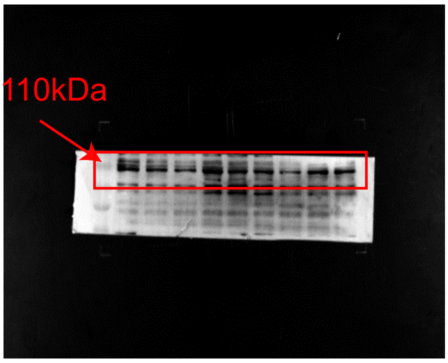

N-cadherin

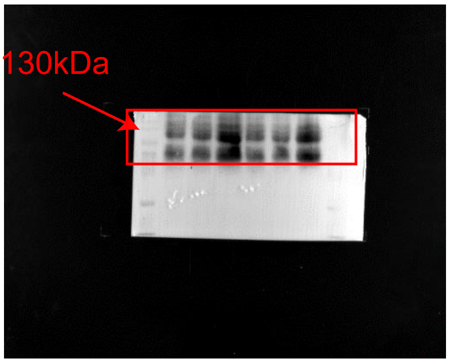

E-cadherin

Fig. 6J

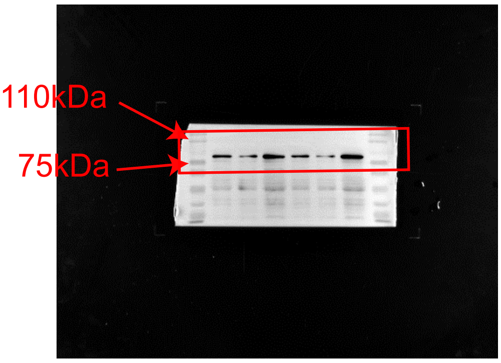

β-catenin

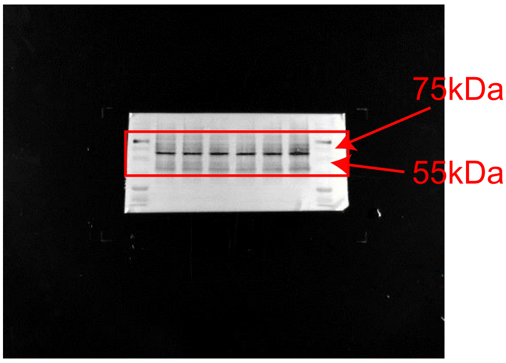

Lamin B1

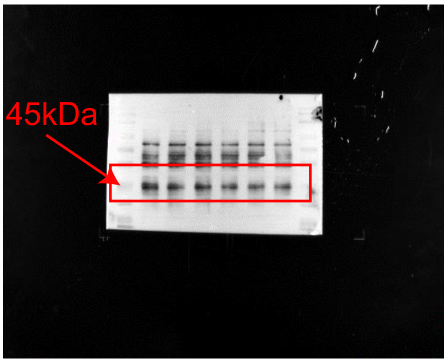

Actin

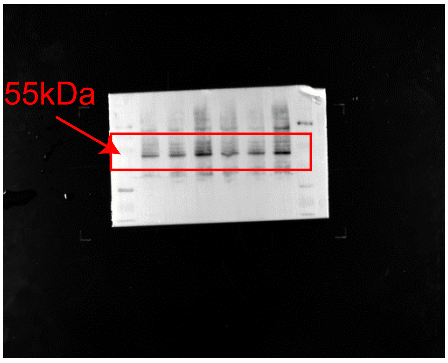

Vim

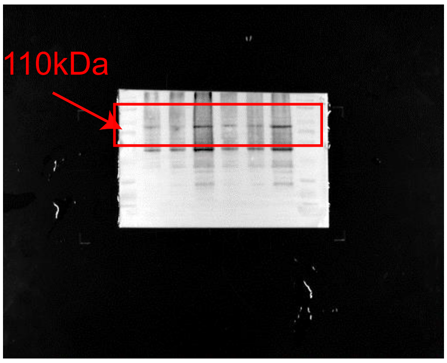

N-cadherin

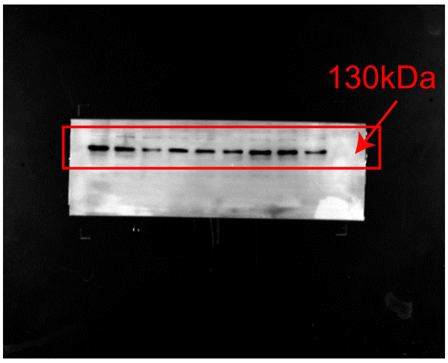

E-cadherin
